# Whole-genome Comparisons Identify Repeated Regulatory Changes Underlying Convergent Appendage Evolution in Diverse Fish Lineages

**DOI:** 10.1093/molbev/msad188

**Published:** 2023-09-21

**Authors:** Heidi I Chen, Yatish Turakhia, Gill Bejerano, David M Kingsley

**Affiliations:** Department of Developmental Biology, Stanford University School of Medicine, Stanford, CA, USA; Department of Electrical and Computer Engineering, University of California, San Diego, CA, USA; Department of Developmental Biology, Stanford University School of Medicine, Stanford, CA, USA; Department of Biomedical Data Science, Stanford University School of Medicine, Stanford, CA, USA; Department of Computer Science, Stanford University School of Engineering, Stanford, CA, USA; Department of Pediatrics, Stanford University School of Medicine, Stanford, CA, USA; Department of Developmental Biology, Stanford University School of Medicine, Stanford, CA, USA; Howard Hughes Medical Institute, Stanford University, Stanford, CA, USA

**Keywords:** repeated evolution, pelvic reduction, genome-wide species comparisons, phenotype-genotype mapping, fish genomics, regulatory deletions

## Abstract

Fins are major functional appendages of fish that have been repeatedly modified in different lineages. To search for genomic changes underlying natural fin diversity, we compared the genomes of 36 percomorph fish species that span over 100 million years of evolution and either have complete or reduced pelvic and caudal fins. We identify 1,614 genomic regions that are well-conserved in fin-complete species but missing from multiple fin-reduced lineages. Recurrent deletions of conserved sequences in wild fin-reduced species are enriched for functions related to appendage development, suggesting that convergent fin reduction at the organismal level is associated with repeated genomic deletions near fin-appendage development genes. We used sequencing and functional enhancer assays to confirm that *PelA*, a *Pitx1* enhancer previously linked to recurrent pelvic loss in sticklebacks, has also been independently deleted and may have contributed to the fin morphology in distantly related pelvic-reduced species. We also identify a novel enhancer that is conserved in the majority of percomorphs, drives caudal fin expression in transgenic stickleback, is missing in tetraodontiform, syngnathid, and synbranchid species with caudal fin reduction, and alters caudal fin development when targeted by genome editing. Our study illustrates a broadly applicable strategy for mapping phenotypes to genotypes across a tree of vertebrate species and highlights notable new examples of regulatory genomic hotspots that have been used to evolve recurrent phenotypes across 100 million years of fish evolution.

## Introduction

Extensive efforts are underway to sequence the genomes of all eukaryotic species (Lewin et al. 2018, 2022)—including roughly 74,000 extant vertebrates (IUCN 2022). Despite dramatic progress in the cost and throughput of whole-genome sequencing, it remains challenging to identify the genomic basis of traits that have evolved repeatedly in wild species. The typical vertebrate genome spans several hundred megabases to several gigabases in length, of which only ∼2% encode proteins (Biscotti et al. 2019; Gregory 2022). Innovative new methods will be needed to compare and interpret genomes in order to identify both the protein-coding and the noncoding regulatory differences that underlie “endless forms most beautiful” that have evolved across the tree of life (Darwin 1859).

Fish species constitute nearly half of all vertebrates, and this enormous radiation exhibits especially remarkable phenotypic diversity (Norman 1949; Nelson, Grande and Wilson 2016; IUCN 2022). A small handful of fishes (e.g., zebrafish, medaka, killifish, stickleback, cichlid) have been developed as useful model organisms for studying vertebrate genetics and development, environmental monitoring, ecological interactions, and evolutionary change (Bell and Foster 1994; Schier and Talbot 2005; Ostlund-Nilsson, Mayer and Huntingford 2006; Katsiadaki et al. 2007; Lleras-Forero, Winkler and Schulte-Merker 2020; Patton, Zon and Langenau 2021; Reid, Bell and Veeramah 2021). Focused studies on these few models have been highly successful, but many more insights will likely come from comparative studies on thousands of additional fish species that have evolved in a diversity of environments.

A useful phenomenon within the teleost radiation includes the abundance of traits that have recurrently evolved across different clades. For example, changes in vertebral number, skeletal armor, teeth, scales, sensory modalities, locomotion (i.e., swimming mechanics, fin “walking,” etc.), osmoregulation, temperature tolerance, and duration of lifespan have evolved multiple times in independent lineages of fish (Norman 1949; Nelson, Grande and Wilson 2016; Kolora et al. 2021). Fin modifications—including extensive loss, dramatic expansion, and/or structural ornamentation—are particularly interesting given the outsized effects of fins on mobility, defense/predation, and reproductive success, and how easily fins can be scored visually or by nondestructive methods (Norman 1949; Davenport 1994; Westneat et al. 2004; Yamanoue, Setiamarga and Matsuura 2010; Price, Friedman and Wainwright 2015; Nelson, Grande and Wilson 2016; Goldberg et al. 2019; Giammona 2021; Sowersby et al. 2022). Because fins are homologous to tetrapod limbs, modification of these major body appendages in fish may also inform a variety of traits and diseases in other animals, including humans (Clack 2009; Tanaka 2016; Larouche et al. 2017, 2019; Letelier et al. 2021; Tzung et al. 2023).

Recent studies suggest that recurrent evolution of phenotypic traits may often take place through reuse of particular genes (Conte et al. 2012; Martin and Orgogozo 2013; Courtier-Orgogozo et al. 2020). As more species’ genomes are sequenced, it may therefore become possible to identify loci controlling certain traits by comparing phenotypes and genotypes over large phylogenetic trees where a trait of interest has evolved multiple times (Smith et al. 2020). This principle has previously been used to identify genomic loci involved in the recurrent evolution of traits as diverse as vitamin C dependence, echolocation, antler loss, flightlessness in birds, and hairlessness in mammals (Hiller et al. 2012a, 2012b; Marcovitz et al. 2019; Sackton et al. 2019; Wang et al. 2019; Kowalczyk et al. 2022). Here, we use a growing number of sequenced fish species to identify genomic regions associated with recurrent fin modifications.

## Results

### A Computational Screen to Identify Genomic Deletions Recurrently Associated With Pelvic Reduction

**Fig. 1.**
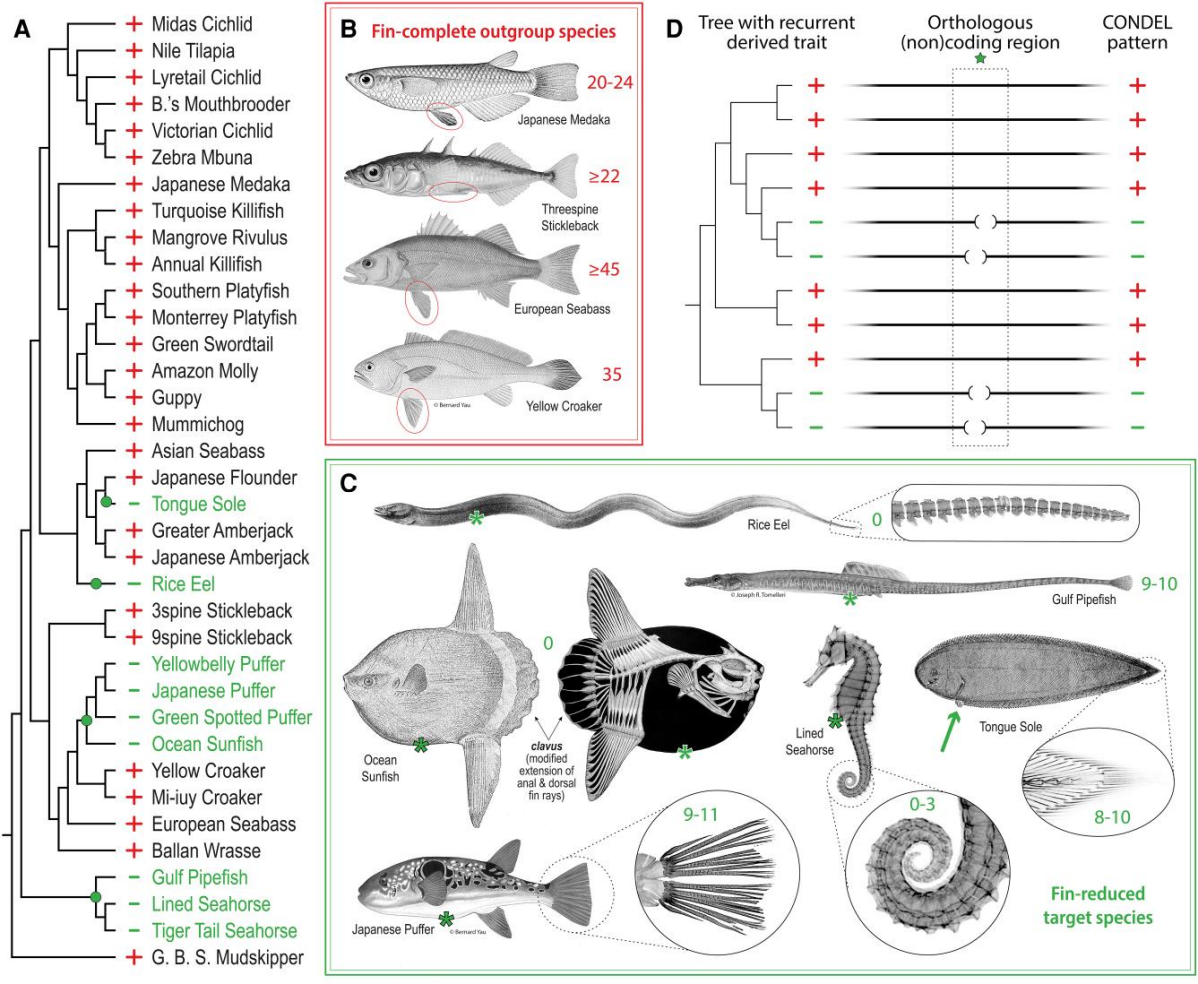
Genome-wide computational screen to identify conserved sequence deletions (CONDELs) associated with fin reduction. (*A*) Pelvic and caudal fin reduction appears to have evolved at least four independent times among the 36 percomorph species examined in this study, as indicated by dots annotated on the phylogenetic tree. Fin-complete “outgroup” species and fin-reduced “target” lineages are denoted with plus (+) and minus (−) signs, respectively. (*B*) Key outgroup species of the study include Japanese Medaka (the reference genome species and origin of the cell line used for in vitro functional experiments), Threespine Stickleback (the species in which all in vivo functional experiments were performed), and European Seabass and Yellow Croaker (additional outgroups to which target lineages were compared in functional studies). All outgroup species exhibit the ancestral state of complete, bilateral pelvic fins (circled) and of complete caudal fins supported by 20 or more bony rays (as indicated by the numerical annotations adjacent to each tail fin). (*C*) Representative species of target lineages exhibiting pelvic and caudal fin reduction include Tongue Sole (of Cynoglossidae); Rice Eel (of Synbranchidae); four members of the order Tetraodontiformes, including Japanese Puffer and Ocean Sunfish; and three members of the family Syngnathidae, including Gulf Pipefish and Lined Seahorse. With the exception of Tongue Sole (which has one pelvic fin on the fish's blind side, thick arrow), all target lineages are pelvic-absent, as indicated by asterisks (*) positioned where pelvic appendages might *once* have existed in the ancestors of these lineages. Rice Eel, Ocean Sunfish, and the two seahorse species exhibit complete or near-complete caudal reduction, as indicated by three or fewer bony rays remaining at their caudal extremes. The tail fins of other target lineages exhibit no more than 11 caudal rays (indicated by the numerical annotations adjacent to the caudal extreme of each species). See supplementary tables S1 and S6, Supplementary Material online, respectively, for references documenting key species phenotypic status and for the sources of illustrations shown in (*B*) and (*C*). (*D*) The genome-wide association strategy implemented in this study requires a tree of broadly related species in which two or more independent sublineages have evolved a recurrent trait. Orthologous whole-genome alignments are then used to focus subsequent experimental interrogation on regions (like the one indicated by a star) with tight correlation between genotype and phenotype—such as the regions of *conserved sequence deletions* (CONDELs) associated with fin reduction identified in the present study.

We selected 36 publicly available, full nuclear genome assemblies that pass a scaffold contiguity criterion of L50 ≤ 300 (see Materials and Methods) and that represent percomorph fish species informative for convergent pelvic fin evolution (fig. 1*A*, see supplementary spreadsheet 1, Supplementary Material online for assembly accession identifiers and sources). Most of the included species have complete bilateral pelvic fins and represent the ancestral, *outgroup* trait status (fig. 1*A* and *B*) (Nelson 1989). In addition, we included representative species from four independent *target* lineages that have evolved dramatic loss of pelvic appendages (fig. 1*A* and *C*; supplementary table S1, Supplementary Material online). The two pelvic-loss clades that were best represented among the assemblies include three members of the family Syngnathidae (pipefish and seahorses) and four members of the order Tetraodontiformes (pufferfishes and Ocean Sunfish). Also included among our target species were one member of the family Synbranchidae (Rice Eel) and one member of the family Cynoglossidae (Tongue Sole, which has one pelvic fin remaining on the blind side of the fish; see supplementary table S1, Supplementary Material online).

Based on overall genome assembly quality, extent of functional annotation, and outgroup trait status, we chose the Japanese Medaka genome assembly (abbreviated oryLat04) to be the reference against which all 35 other query genome sequences were aligned (Ichikawa et al. 2017). Using only orthology-confident alignment chains, we then searched for genomic sequences that were highly conserved in most outgroup species that had intact pelvic fins but were missing (deleted or extensively diverged) in multiple syngnathid and tetraodontiform target species that showed pelvic loss (see fig. 1*D* and Materials and Methods). We termed these regions *percomorph conserved sequence deletions* or *pCONDELs* and reasoned that some of these candidate intervals are likely involved in the control of pelvic fin development. Because multiple species in Synbranchidae and Cynoglossidae were not able to be included, a genotype–phenotype match in these single-representative target clades was not strictly required in the computational screen but was considered in selecting candidates for subsequent functional experiments.

With these criteria, we scanned intervals spanning 200 kb upstream to 200 kb downstream of the transcription start site for all successfully mapped reference gene orthologs. In total, we identified 1,614 predicted conserved sequences that were missing in multiple individuals of at least two independent target clades with pelvic reduction (see Materials and Methods). Of these pCONDELs, 9.5% intersected protein-coding exons, 49.6% intersected noncoding regions within genes, and 40.9% were located in intergenic regions of the Japanese Medaka reference assembly (see supplementary spreadsheet 2, Supplementary Material online). Notably, the 3,489 gene orthologs that were linked to these candidate regions were most enriched for functions related to *medial fin development* (GO:0033338, 4.45-fold enrichment, *q*-value = 0.022) and *embryonic appendage morphogenesis* (GO:0035113, 3.63-fold enrichment, *q*-value = 0.026). These two most-enriched ontology terms suggest that convergent fin loss at the phenotypic level is associated with repeated genomic deletions that remove conserved sequences near fin and appendage development genes, including *Acvr1l, Bmp4, Dlx6a, Ext2, Extl3, Fgf10a, Fndc3a, Hmcn2, Lef1, Rspo2, Sall4, Shha, Smo, Sox9, Sp9, Tbx5a, and Wnt2ba* (see supplementary spreadsheets 2 and 3, Supplementary Material online for the full list of genes linked to pCONDELs as well as all enriched ontology terms).

For an indication of pCONDEL age, we used BLAST and chained LASTZ whole-genome alignments to identify sequences that were likely already present in the common ancestor of Japanese Medaka and the nonpercomorph Zebrafish (see Materials and Methods). We manually inspected each homology candidate using UCSC Genome Browser visualizations for oryLat04 and danRer11 (Kent et al. 2002; Raney et al. 2014) and determined that at least ∼18% (*n* = 286/1,614, see column “conserved to zebrafish” in supplementary spreadsheet 2, Supplementary Material online) of the pCONDEL regions have sequence homologs that were present in the most recent common actinopterygian ancestor of these taxa, which existed ∼240 million years ago (Kumar et al. 2022). Of the 286 actinopterygian-conserved pCONDELs, 140 intersected protein-coding exons, 69 intersected noncoding regions within genes, and 77 were located in intergenic regions of the Japanese Medaka reference assembly (see supplementary spreadsheet 2, Supplementary Material online).

### Recurrent Deletions at the *PelA* Pelvic Enhancer of *Pitx1*

**Fig. 2.**
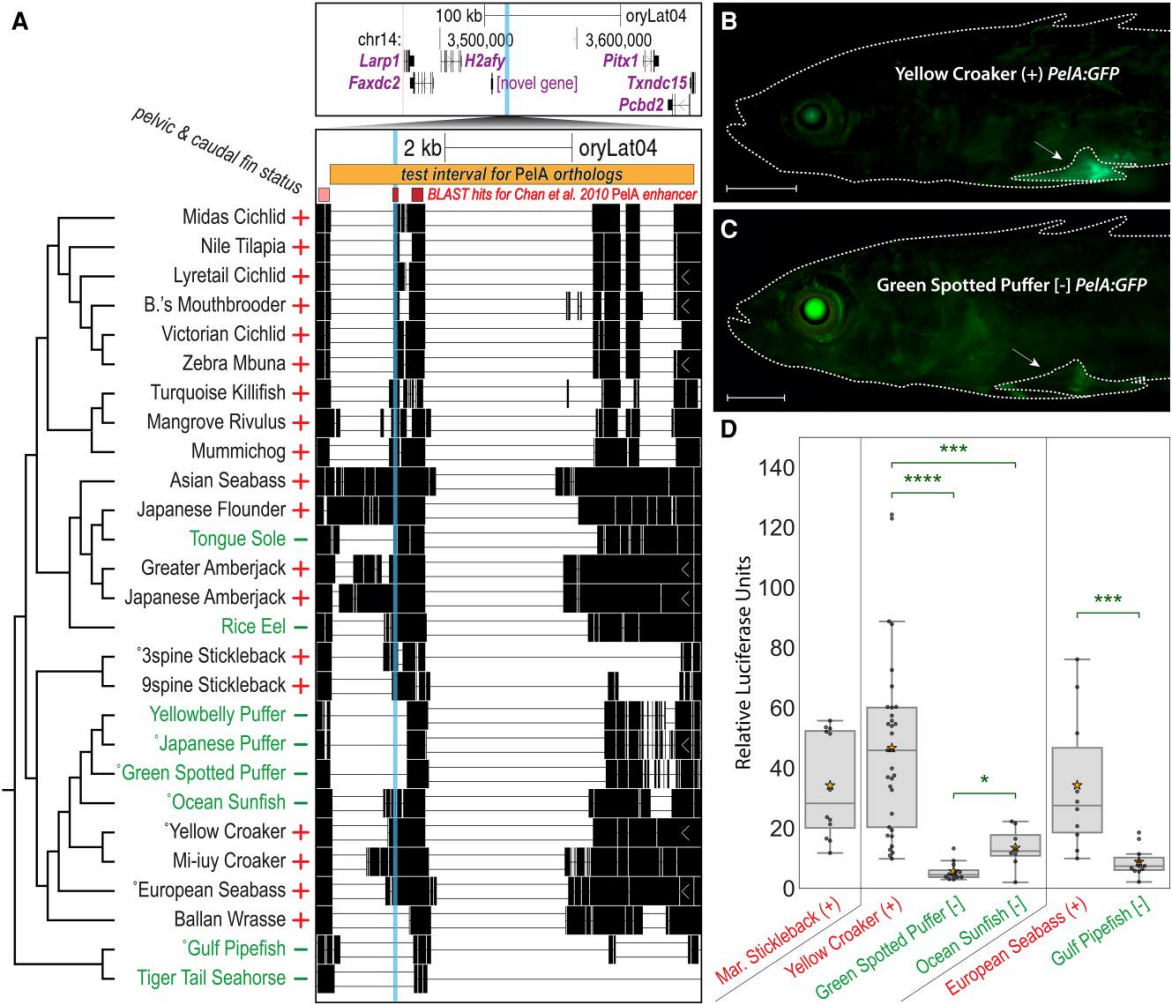
Recurrent deletions in multiple fish species reduce functional activity of the *PelA* enhancer. (*A*) The 50 bp candidate interval pCONDEL.329 (vertical cyan highlight) is located ∼100 kb upstream of the hind appendage control gene *Pitx1* (top genome browser view), a region orthologous to the *PelA* pelvic enhancer of the Threespine Stickleback *Pitx1* gene (Chan et al. 2010; Xie et al. 2019). Regions of BLAST similarity to the 2.5 kb Threespine Stickleback *PelA* sequence are shown in an annotation track as shaded (pink and red) bars. The darker (red) bars overlap pCONDEL.329 and denote BLAST hits specifically to the 500 bp core functional element of *PelA*. All existing pairwise orthologous alignment chains mapped to *Pitx1* are shown. The bar labeled “*test interval for* PelA *orthologs*” (oryLat04.chr14:3,547,836–3,553,572) marks the interval of orthologous sequences tested in the subsequent functional experiments depicted in (*B*–*D*) and/or verified by Sanger sequencing from the indicated (˚) target and outgroup species. Plus (+) and minus (−) signs denote phenotypic trait status (presence or absence of pelvic and caudal fin reduction). See supplementary figure S2, Supplementary Material online for the nucleotide-level multiple sequence alignment of pCONDEL.329. (*B, C*) GFP reporter expression in transgenic Threespine Stickleback driven by *PelA* orthologs from pelvic-complete (+) Yellow Croaker (*B*) and pelvic-absent [−] Green Spotted Puffer (*C*). Scale bars are 1 mm. White arrows point to the developing left pelvic girdle and spine. (*D*) Relative luciferase expression in cultured fin cells driven by *PelA* orthologs from: pelvic-complete Marine Threespine Stickleback; pelvic-reduced Green Spotted Puffer and Ocean Sunfish and their nearest pelvic-complete outgroup Yellow Croaker; and pelvic-reduced Gulf Pipefish and its nearest pelvic-complete outgroup European Seabass. Boxes show quartiles; stars denote the mean of all points in the category. Two-tailed Mann–Whitney *U* test *P*-values: *, < 0.02; ***, < 4*e*−3; ****, < 1*e*−6.

One of the regions identified by the computational screen for recurrent deletions in pelvic-less percomorph clades (pCONDEL.329) is a 50 bp interval that exhibits BLAST sequence similarity to the *PelA* pelvic enhancer of the limb and pituitary development gene *Pitx1*. In particular, pCONDEL.329 appears to be orthologous specifically to part of the minimal 500 bp core functional element of the *PelA* enhancer sequence (fig. 2*A*) (Chan et al. 2010). Previous studies found that recurrent pelvic reduction in multiple freshwater populations of Threespine Stickleback is largely attributable to repeated, de novo deletion of this noncoding pelvic control region (Shapiro et al. 2004; Chan et al. 2010; Xie et al. 2019). As in stickleback, the Japanese Medaka ortholog of the *PelA* enhancer is also upstream of the *Pitx1* gene. To confirm the presence of *PelA* deletions in other pelvic-reduced lineages, we used PCR to amplify across the orthologous region from relevant target species and their nearest outgroup (see Materials and Methods, supplementary fig. S1, text 1, and file 4, Supplementary Material online). Sanger sequencing confirmed that most pelvic-reduced species show independent deletions of different sizes and breakpoints as well as varied nucleotide substitutions (see Materials and Methods, GenBank accession numbers in supplementary table S2 and fig. S2, Supplementary Material online), resulting in the loss of at least 78 bp of the 500 bp core *PelA* enhancer (corresponding to gasAcu1-4.chrP:129,576–129,653 in the Threespine Stickleback reference genome).

To test whether these natural deletions also altered *PelA* function, we amplified the orthologous sequences denoted by the orange bar in figure 2*A* from several key species, cloned them upstream of a minimal promoter and GFP reporter gene, and injected the constructs into fertilized eggs from pelvic-complete Threespine Stickleback. The *PelA* sequence from outgroup Yellow Croaker drove bright GFP expression in developing pelvic structures of transgenic larvae (fig. 2*B*, *n* = 11/16 fish from two clutches), in patterns similar to that of the previously characterized marine stickleback ortholog (*n* = 11/18 fish from 1 clutch, data not shown) (Chan et al. 2010). Only weak and diffuse pelvic expression was seen from the cloned *PelA* region of the pelvic-reduced Green Spotted Puffer (fig. 2*C*, *n* = 7/10 fish from 3 clutches), and this expression appeared substantially reduced compared to control eye expression known to occur from the *hsp70* minimal promoter of the transgenic construct (fig. 2*B* and *C*) (Nagayoshi et al. 2008).

For a more quantitative assessment of the activity of various *PelA* orthologs, we cloned these regions upstream of a luciferase reporter gene and compared levels of luciferase activity following transfection into OLHNI-2 cultured fin cells derived from Japanese Medaka (Hirayama, Mitani and Watabe 2006). Sequence from the previously characterized marine stickleback *PelA* region drove substantial expression in cultured fin cells, as did sequences from pelvic-complete outgroup species such as Yellow Croaker and European Sea Bass. In contrast, orthologs from two different pelvic-absent tetraodontiform species (Ocean Sunfish and Green Spotted Puffer), and from pelvic-absent Gulf Pipefish, drove significantly weaker luciferase activity compared to their nearest pelvic-complete outgroup (fig. 2*D*). Notably, the relative strengths of the *PelA* alleles from Ocean Sunfish and Green Spotted Puffer (both pelvic-absent) were inversely correlated with the amount of sequence (containing putative transcription factor binding sites) deleted in this region (supplementary fig. S2, Supplementary Material online). Previous studies have associated repeated deletions of *PelA* with recurrent pelvic reduction occurring over the last 10,000–20,000 years of stickleback evolution (Shapiro et al. 2004; Chan et al. 2010; Xie et al. 2019). Our results identify similar deletions also operating over a significantly wider, 100+ million-year span of fish evolution (Kumar et al. 2022), and confirm that our whole-genome comparative screen can identify functionally important lesions in known pelvic development loci.

### A Novel, Recurrently Deleted Regulatory Region Near *Faf1*

**Fig. 3.**
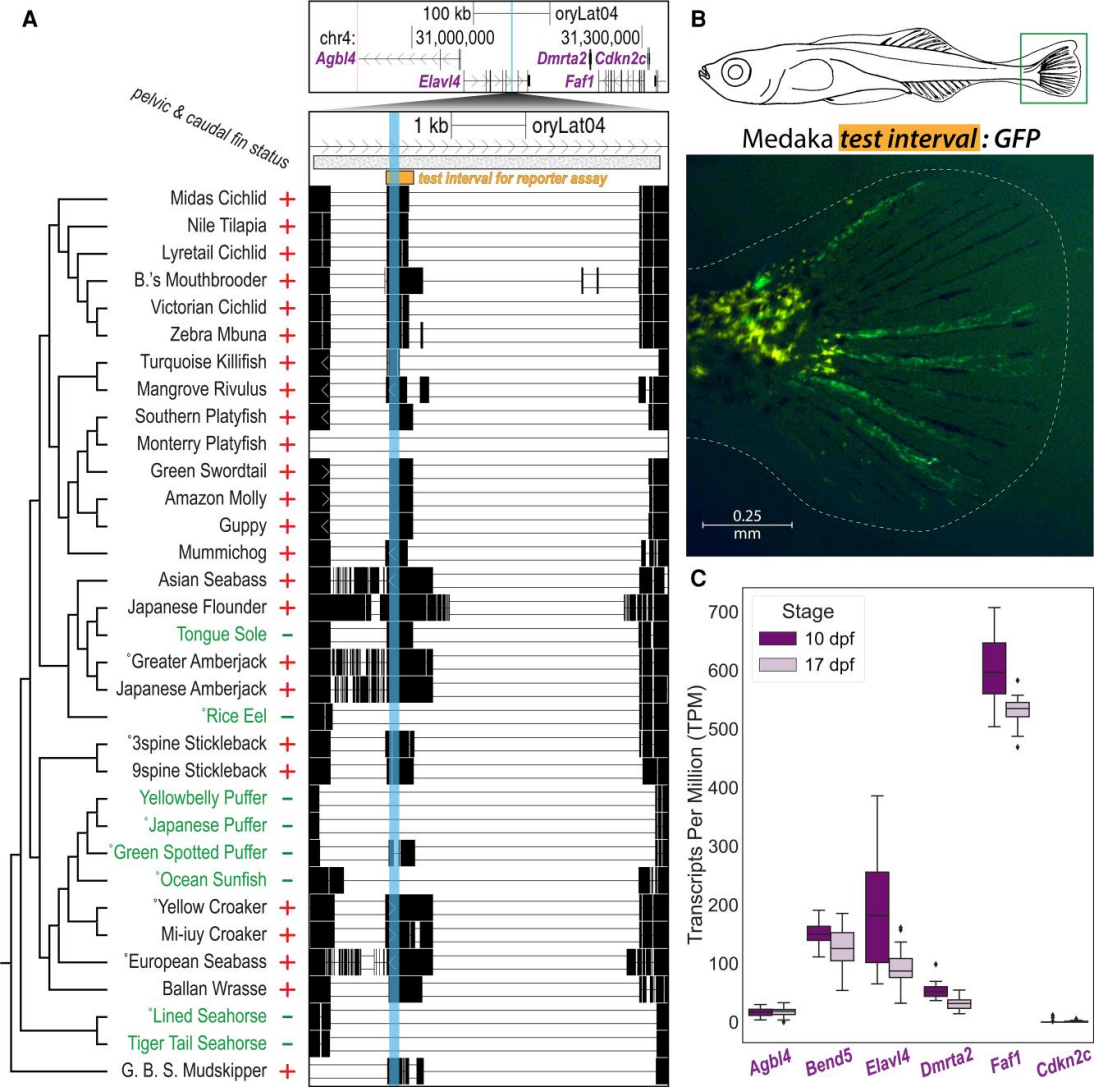
A novel, recurrently deleted regulatory region near *Faf1*. (*A*) The 135 bp candidate pCONDEL.1189 (vertical cyan highlight ) is located within an intron of *Elavl4*. The top genome browser view shows a region spanning 200 kb on both sides of pCONDEL.1189 (Japanese Medaka sequence space). Pairwise alignment chains for all species with orthologous mappings to *Elavl4* and *Faf1* are shown in the bottom genome browser view. The bar labeled “*test interval for reporter assay*” (oryLat04.chr4:31,129,554–31,130,065) marks the interval of orthologous sequence from Japanese Medaka tested in (*B*). The hatched bar (oryLat04.chr4:31,128,601–31,133,355) marks the interval verified by Sanger sequencing from the indicated (˚) target and outgroup species orthologs. Plus (+) and minus (−) signs denote phenotypic trait status (presence or absence of pelvic and caudal fin reduction). See supplementary figure S3, Supplementary Material online for the nucleotide-level multiple sequence alignment of pCONDEL.1189. (*B*) GFP reporter expression in the caudal fin (demarcated with a box) of a transgenic Threespine Stickleback larva at Swarup stage 30 (schematic modified from Swarup 1958) driven by the test interval in (*A*) cloned from Japanese Medaka genomic DNA. (*C*) Normalized gene expression data in the developing caudal fins of Threespine Stickleback (*n* = 12 at each stage, before and after fin ray development) for all orthologous genes with transcription start sites predicted to lie within 200 kb of pCONDEL.1189 as shown in (*A*). Data for *Bend5*—a gene between *Agbl4* and *Elavl4* found in Threespine Stickleback but not in Japanese Medaka—are also included. *dpf,* days post fertilization.

To study a completely novel region identified by the screen, we chose to explore pCONDEL.1189, whose presence or absence was even more tightly correlated with pelvic fin status than *PelA.* Located in an intron of *Elavl4*, this 135 bp region appeared to be either missing or disrupted in all scorable syngnathids and tetraodontiforms as well as in Rice Eel (fig. 3*A* and supplementary fig. S3, Supplementary Material online). We again confirmed the computational predictions in this region by amplifying and sequencing across the genomic interval pictured in figure 3*A* from relevant target species and their nearest outgroups (see Materials and Methods, GenBank accession numbers in supplementary table S2, Supplementary Material online).

Given the absence of previous functional information about this candidate interval, we tested whether the conserved sequence element could drive informative GFP reporter expression in transgenic assays of enhancer activity in vivo. To do this, we cloned a ∼500 bp test interval (as indicated by the orange bar in fig. 3*A*) from Japanese Medaka genomic DNA upstream of a transposable GFP reporter and injected the construct into fertilized eggs of pelvic-complete Threespine Stickleback. Although we hypothesized that we would see enhancer activity in the developing pelvis of transgenic larvae, we instead observed consistent reporter expression in the developing caudal fin (fig. 3*B*, *n* = 15/21 transgenic fish from six clutches). These results suggested the candidate region might actually be a tail fin enhancer that was lost in many of the target lineages. A review of published literature and publicly accessible radiographs showed that the pelvic-reduced species in our study also exhibited varying and significant degrees of reduction in the caudal fin (Larouche et al. 2017). Pelvic-complete outgroup species showed totals of 20 or more segmented plus *un*segmented bony fin rays (lepidotrichia) present in their caudal fins (see numerical annotations in fig. 1*B* and references in supplementary table S1, Supplementary Material online). In contrast, pelvic-reduced species in our screen showed totals of 11 or fewer caudal fin rays (see numerical annotations in fig. 1*C*; significance of change in number of caudal fin rays determined by two-tailed Welch's test: *t* = 5.64, df = 6.42, *P* = 1.16e−3). Note that all ecotypes of adult Threespine Stickleback possess 22 or more lepidotrichia (supplementary fig. S4, Supplementary Material online and see Plate III of the study published by Huxley), making this species an outgroup in terms of caudal fin status (Huxley 1859; Lindsey 1962).

Given the caudal fin enhancer activity of the pCONDEL.1189 region, we performed RNAseq to determine whether any genes in the surrounding genomic interval were also expressed in the developing caudal fins of Threespine Stickleback fry before and during lepidotrichia formation (10 and 17 days post fertilization, respectively) (Swarup 1958). Of the five genes with transcription start sites within 200 kb of pCONDEL.1189 in the Japanese Medaka genome assembly, *FAS-associated factor 1* (*Faf1*) was most highly expressed (fig. 3*A* and *C*). *Faf1* encodes a protein that associates with the cell death receptor FAS (TNFRSF6) and has been shown to modulate apoptosis and cell proliferation, including in skeletal tissues (see Discussion).

### Morphological Effects of Engineered Mutations in pCONDEL.1189 and *Faf1*

**Fig. 4.**
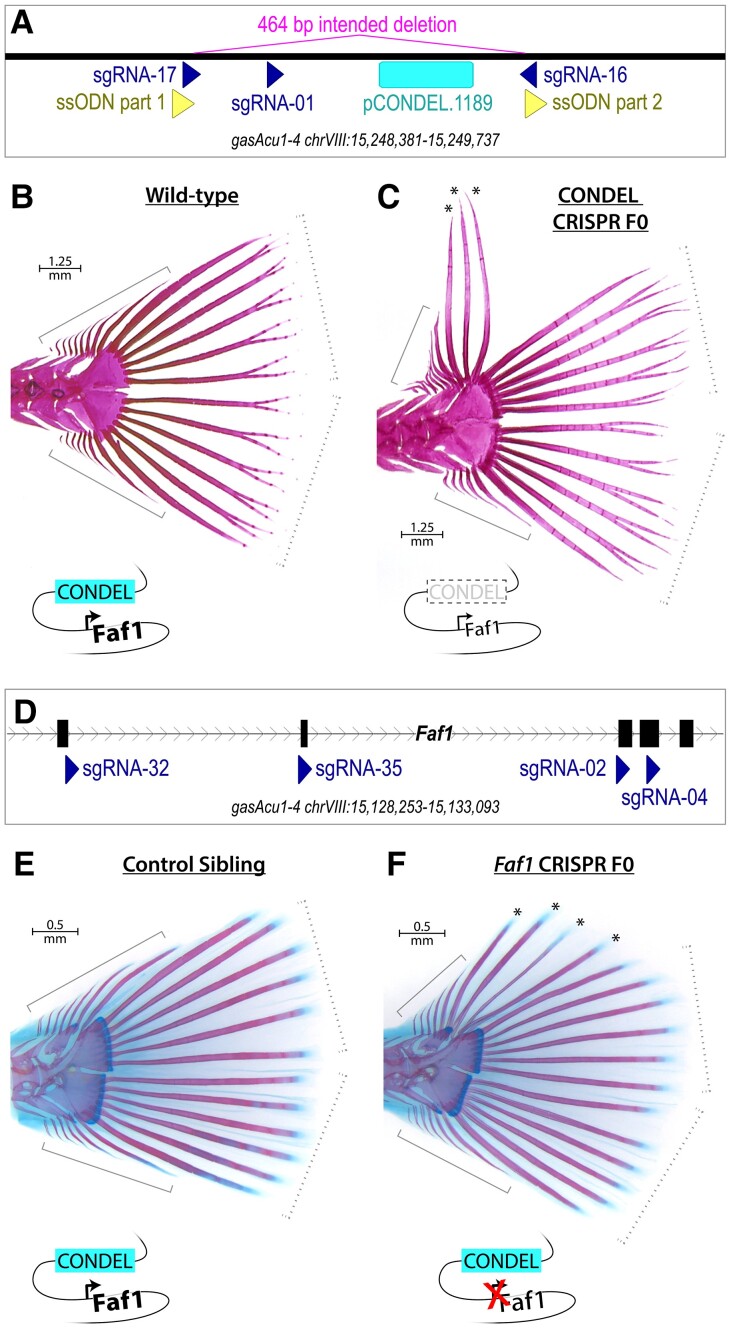
Morphological effects of engineered mutations in pCONDEL.1189 and *Faf1*. (*A*) The conserved element at pCONDEL.1189 (labeled bar) was deleted from Threespine Stickleback by injecting zygotes with Cas9 protein, three sgRNAs (darkly shaded triangles), and a 60 bp single-stranded DNA oligonucleotide (*ssODN*, a concatemer of the two lightly shaded triangles) to promote homology-directed repair and creation of the indicated 464 bp deletion. (*B*) Alizarin red-stained wild-type Threespine Stickleback exhibiting the typical six segmented *principal* rays on each of the dorsal and ventral halves of the tail fin (dashed brackets) as well as a variable number of smaller, unsegmented *simple* rays more anteriorly (solid brackets). (*C*) Alizarin red-stained mosaic F0 crispant targeted for deletion at the pCONDEL.1189 region showing additional long segmented rays (*) where typically only unsegmented simple rays exist. (*D*) *Faf1*, putative target gene of the conserved enhancer at pCONDEL.1189, was disrupted by injecting Threespine Stickleback zygotes with Cas9 protein and four sgRNAs (darkly shaded triangles) targeting exons 2–5 of the gene (furthest right exon 6 is also pictured). (*E*) Alizarin red and alcian blue-stained unmodified control sibling exhibiting the typical six segmented *principal* rays on each of the dorsal and ventral halves of the tail fin (dashed brackets) as well as a variable number of smaller, unsegmented *simple* rays more anteriorly (solid brackets). (*F*) Alizarin red and alcian blue-stained fish targeted for inactivation of *Faf1* showing ectopic segmented rays (*) where typically only unsegmented simple rays exist. Schematics summarizing the genome modifications performed near pCONDEL.1189 and *Faf1*, as well as their hypothesized mechanisms of action, are included below (*B, C*) and (*E, F*).

To test whether pCONDEL.1189 was required for normal caudal fin development, we used CRISPR-Cas9 editing in Threespine Stickleback to remove the conserved sequence from an outgroup species that normally exhibits complete caudal and pelvic fin development (fig. 4*A*). Following injections of Cas9 and single-guide RNAs (sgRNAs) into fertilized stickleback eggs, genotyping and sequencing confirmed high efficiency removal of the conserved element in resulting fish (supplementary fig. S5*A*–*C*, Supplementary Material online). Across 17 genome-edited clutches, 8% of the pCONDEL.1189-targeted fish (*n* = 14/175) exhibited ectopic overgrowth of the typically unsegmented procurrent rays on the dorsal edge of the tail (fig. 4*B* and *C*; supplementary figs. S5*D*and S6, Supplementary Material online). To control for potentially confounding effects of Cas9 nuclease injection at the zygote stage, we simultaneously injected clutch siblings with equal concentrations of editing reagents (see supplementary table S3, Supplementary Material online) that instead targeted the previously characterized *Slc24a5* gene of the *golden* pigment-control locus (Lamason et al. 2005; Wucherpfennig, Miller and Kingsley 2019). Only a single *golden* knockout control sibling also showed tail abnormalities (0.43%, *n* = 1/236, supplementary fig. S6, Supplementary Material online), confirming a significant effect of pCONDEL.1189 targeting on caudal fin development (two-tailed Boschloo's Exact Test: statistic = 4.40e−5, *P* = 3.55e−5). In three clutches generated by intercrossing pCONDEL.1189 mosaic founder fish, no mutant tail fin phenotypes were seen, even in offspring missing both copies of the conserved sequence at the candidate region (*n* = 85; see supplementary fig. S7, Supplementary Material online and additional discussion below).

To test whether the hypothesized target gene, *Faf1*, was similarly required for normal caudal fin development, we also injected Threespine Stickleback zygotes with a pool of 4 sgRNAs targeting early exons of the gene (fig. 4*D*). Sequencing confirmed the creation of indels disrupting *Faf1* coding exons in resulting fish (supplementary fig. S8*A*–*C*, Supplementary Material online). Across 10 genome-edited clutches, we observed that 6% of fish (*n* = 16/265) exhibited a phenotype very similar to that of the pCONDEL.1189 enhancer deletion fish, with unusually long and segmented rays on the dorsal edge of the tail (fig. 4*E* and *F*; supplementary figs. S8*D*and S9, Supplementary Material online). No mutant tail fin phenotypes were seen in 277 unmodified control siblings from the same clutches, confirming a significant effect of *Faf1* targeting on caudal fin development (two-tailed Boschloo's Exact Test: statistic = 8.40e−6, *P* = 6.66e−6).

## Discussion

Previous studies have identified “hotspots of evolution”—particular genomic regions that are used repeatedly when similar traits evolved in multiple lineages across the tree of life (Martin and Orgogozo 2013). Over 115 hotspot loci have been cataloged that show both computational and functional experimental evidence of having been used repeatedly during independent evolution of traits across closely related populations within a species (intraspecific hotspots). In contrast, about 4-fold fewer loci are currently known that underlie repeated evolution across higher taxonomic levels (i.e., hotspots reused between individuals of different genera and even more distant phylogenetic taxa) (Courtier-Orgogozo et al. 2020).

Extensive linkage mapping, mutation, transgenic, and genome-editing studies have previously shown that the *PelA* enhancer of the *Pitx1* gene corresponds to an intraspecific genomic hotspot for repeated pelvic loss within Threespine Stickleback (*Gasterosteus aculeatus*) (Shapiro et al. 2004; Chan et al. 2010; Xie et al. 2019). Multiple *Gasterosteus* populations have independently evolved total loss of pelvic fins in new freshwater lakes generated by widespread melting of glaciers in the last 20,000 years. The mutation spectrum in these pelvic-reduced *Gasterosteus* populations consists of deletions of a few hundred to a few thousand bases that completely eliminate a shared 369 bp region of the *PelA* pelvic enhancer control region (Chan et al. 2010; Xie et al. 2019). Pelvic reduction can be phenocopied by targeted deletions of the *PelA* region (Xie et al. 2019), or rescued by reintroducing *PelA*:*Pitx1* constructs into pelvic-less fish (Chan et al. 2010), confirming that *PelA* is a key causal locus for loss of pelvic structures within the *Gasterosteus aculeatus* species complex.

Genetic studies also suggest that changes in the *Pitx1* locus underlie some examples of pelvic reduction in the more distantly related Ninespine Stickleback lineage (*Pungitius pungitius*) (Shapiro et al. 2009; Shikano et al. 2013; Kemppainen et al. 2021). However, the genetic basis of pelvic reduction appears to be heterogeneous across different *Pungitius* populations, and the causative molecular lesions in most populations are still unknown (Shapiro et al. 2009; Shikano et al. 2013; Kemppainen et al. 2021).

Our current study has identified recurrent deletions of the *PelA* enhancer occurring in distantly related wild fish species that have independently evolved pelvic reduction since their divergence over 100 million years ago. These results show that the *PelA* locus is not only an intraspecific evolutionary hotspot but also an interordinal hotspot that is reused when pelvic reduction evolves in different orders of fishes. Most previous examples of hotspot loci reused between distant phylogenetic groups and cataloged in “GePheBase: The Database of Genotype-Phenotype Relationships” have involved repeated amino acid changes in particular proteins (Courtier-Orgogozo et al. 2020). *PelA* is one of a very few experimentally validated *cis*-regulatory regions now known to underlie naturally evolving traits that have converged in species belonging to separate genera, families, orders, or phyla (Sagai et al. 2004; Miller et al. 2007; Guerreiro et al. 2013; Kvon et al. 2016; Sackton et al. 2019; Courtier-Orgogozo et al. 2020; Wucherpfennig et al. 2022).

Although the *PelA* region had previously been implicated in pelvic reduction, most of the other genomic regions recovered in our genome-wide comparative analysis do not have previously known functions. Over 90% map outside of protein-coding exons of genes, so, like *PelA*, may also correspond to regulatory elements in the genome. The genomic regions near these recurrently lost sequences are enriched for genes involved in fin and appendage development, and we hypothesize that regulatory deletions occurring near such genes are an important contributor to recurrent fin evolution in fishes.

Our studies clearly illustrate the value of experimental validation of novel loci identified by comparative genomic scans. Although we selected target and outgroup species to screen for regions associated with pelvic reduction, when we tested the in vivo activity of the conserved element at pCONDEL.1189, we found that this noncoding regulatory region drove expression in caudal fins rather than pelvic fins of transgenic larvae. Retrospective trait inspection showed that pelvic *and* caudal fin reduction are correlated with each other in the original species tree. Thus, the genomic regions recovered in our whole-genome screen might be associated with either of these fin phenotypes or with any other trait (such as branchial arch modification) that co-occurs with pelvic and caudal fin reduction in the same target lineages (Larouche et al. 2017). We have not yet experimentally tested other regions from the computational screen for in vivo expression patterns and phenotypes, so do not know what fraction of pCONDELs may regulate expression in pelvic fins, median fins, or other tissues. Future surveys characterizing tissue-specific patterns of chromatin accessibility might aid in discerning which tissue(s) and trait(s) are most likely associated with particular pCONDELs identified in the computational screen (e.g., Sackton et al. 2019).

Our genome-editing experiments further confirm that the pCONDEL.1189 region functions during caudal fin development. Deletion of the region results in the formation of additional long segmented fin rays in the caudal fins of Threespine Stickleback. Targeting of the nearby *Faf1* gene produces a very similar phenotype, suggesting the pCONDEL.1189 region likely acts by regulating *Faf1* function. *Faf1* is named for its association with the *Fas1* receptor, which activates cell death pathways in response to intercellular signaling (Chu, Niu and Williams 1995; Ryu et al. 2003). The extra fin rays seen in our targeting experiments may thus be the result of decreasing the activity of a known apoptotic pathway during caudal fin development. Since recurrent loss of the region is correlated with recurrent reduction of fin rays in natural fish species, we speculate that evolution of caudal fin morphology may involve a mixture of both reductive and compensatory mutations.

It is interesting that tail phenotypes were only observed in a fraction of CRISPR/Cas9-edited founder fish: approximately 8% of pCONDEL.1189-targeted fish and 6% of *Faf1*-targeted fish (supplementary figs. S5*D*and S8*D*, Supplementary Material online), versus ≥50% of founder fish exhibiting obvious phenotypes when targeted at previously reported pigmentation loci (Wucherpfennig, Miller and Kingsley 2019). In addition, we did not observe similar tail phenotypes when we intercrossed mosaic founders to produce F1 fish that were homozygous for mutant pCONDEL.1189 alleles in every cell during normal development (supplementary fig. S7, Supplementary Material online). These results might be attributable to the polar coordinate model of limb development first described by French, Bryant and Bryant (1976). Based on observations of supernumerary limb and digit formation in insects and amphibians after mirror-image appendage grafting, this model posits that every cell has a particular positional identity relative to all other cells in a given tissue, as if arranged in a polar coordinate system. When normally nonadjacent identities come into contact, either by injury, tissue grafting, or—we suggest—mosaic genome editing, cells proliferate and/or intercalate to restore the original cell–cell adjacencies and thereby generate supernumerary structures. We hypothesize that the ectopic fin ray phenotype in our mosaic founders may occur when *Faf1* activity is disrupted in subsets of developing fin cells, and perhaps only when those cells occur in juxtaposition with other cells that have different relative levels of *Faf1* activity. Future studies could modulate *Fas1*/*Faf1* activity in subgroups of cells during caudal fin development to test whether manipulation of this pathway in specific anatomical regions leads to predictable morphology that resembles fin ray changes in wild fishes.

Our current work on *PelA* and pCONDEL.1189 illustrates how genome-wide approaches can identify both known and novel loci contributing to repeated evolution of fish fin modifications. In our genomic comparisons, we focused on a specific type of convergent DNA change (deletions), because such structural changes seemed likely to yield phenotypic consequences and were already associated with repeated evolution of pelvic reduction in multiple stickleback populations (Chan et al. 2010; Xie et al. 2019). However, we note that similar genome-wide association approaches can be designed to identify many other types of DNA alterations, which likely differ in degree of pleiotropy and selective constraints (e.g., regions of accelerated base pair substitutions, convergent amino acid changes, transcription factor binding site modifications, splicing changes, insertions, copy number differences, or multiple types of lesions co-occurring in the same gene or gene pathway in different lineages (Hiller et al. 2012a, 2012b; Chikina, Robinson and Clark 2016; Marcovitz, Jia and Bejerano 2016; Prudent et al. 2016; Lowe et al. 2017; Partha et al. 2017; Berger et al. 2018; Marcovitz et al. 2019; Sackton et al. 2019; He et al. 2020; Kowalczyk et al. 2020; Turakhia et al. 2020; Kaplow et al. 2022; Kowalczyk, Chikina and Clark 2022; Roscito et al. 2022)). We also note that our screen used a comparative genomic analysis that was anchored to a particular reference species assembly. This strategy will miss potential candidate regions which are not present in the reference assembly itself (due to either biological or artifactual reasons), and we encourage future studies to extend our efforts by using reference-free multiple sequence alignments, such as those generated by Progressive Cactus (Armstrong et al. 2020).

It is still not clear how often repeated phenotypic evolution is due to convergent mutational changes in the same genomic regions (Gompel and Prud’homme 2009; Conte et al. 2012; Martin and Orgogozo 2013). Recent reviews of known cases suggest the answer may vary depending on the phylogenetic distance between lineages in which convergent phenotypes arise, with more closely related species being most likely to evolve through similar genetic pathways (Conte et al. 2012; Ord and Summers 2015). On the other hand, reuse of certain genomic loci still appears to be appreciable even among distantly related lineages (Conte et al. 2012; Martin and Orgogozo 2013; Courtier-Orgogozo et al. 2020), and our results provide striking new examples of genomic convergence occurring across fish species that last shared a common ancestor over 100 million years ago. Although we focused on fin modifications in this study, many other types of traits also evolve repeatedly when different lineages adapt to similar ecological pressures (McGhee 2011). As fish make up nearly half of vertebrate species and also account for a large fraction of known examples of repeated evolution (Ord and Summers 2015), they may provide an especially powerful system for linking phenotypes and genotypes using tree-wide association and functional genomic studies.

## Materials and Methods

### Computational Screen to Identify Conserved Sequence Deletions

#### Genome Assemblies

See supplementary spreadsheet 1, Supplementary Material online for accession identifiers and sources. We required assemblies used in the screen to have a scaffold L50 ≤ 300. The scaffold L50 statistic is defined as the smallest number of scaffolds whose lengths sum to half of the total genome size. Unlike the N50 statistic, it is normalized to genome size and is thus more informative when assessing the relative quality of different species’ (variably sized) genome assemblies.

#### Repeat Masking

For assemblies hipEre01 and synSco01, RepeatMasker v4.1.0 (with NCBI/RMBLAST [2.10.0+] and Complete Master RepeatMasker DatabaseCONS-Dfam_3.1-rb20181026) was used to soft-mask repetitive sequences prior to whole-genome alignment (Smit, Hubley and Green 2015). Specifically, the following commands were run:

RepeatMasker -engine rmblast -species “hippocampus erectus” -s -no_is -cutoff 255 -frag 20000 hipEre01.fa

RepeatMasker -engine rmblast -species “syngnathus scovelli” -s -no_is -cutoff 255 -frag 20000 synSco01.fa

#### Whole-genome Alignment and Orthology Mapping

Based on its high level of contiguity and functional annotation, the Japanese Medaka genome assembly ASM223467v1 (GenBank accession # GCA_002234675.1, assembly abbreviation oryLat04) was used as the reference to which all 35 other percomorph query genome sequences were aligned (Ichikawa et al. 2017). Ensembl release 98 gene models for ASM223467v1 served as the genomic landmarks for identifying orthologous alignment chains (Cunningham et al. 2022). LASTZ-based pairwise whole-genome alignment chains (Kent et al. 2003) were generated using the doBlastzChainNet.pl tool (https://github.com/ENCODE-DCC/kentUtils/, last accessed 12 Sep 2022) and the parameters listed in supplementary spreadsheet 1, Supplementary Material online. The chainLinearGap parameter was set to loose for alignment to the nonpercomorph Zebrafish assembly (danRer11), and to medium for all other percomorph genome alignments. Only alignment chains containing confident gene ortholog mappings, identified as previously described (Turakhia et al. 2020) with gene-in-synteny and second-best-chain-ratio thresholds of 10, were used in subsequent computational steps to identify conserved sequence deletions (CONDELs). All orthologous pairwise alignment chains can be viewed at the following UCSC Genome Browser assembly hub (Kent et al. 2002; Raney et al. 2014): https://genome.ucsc.edu/s/hchen17/oryLat04_public.

#### Identifying Conserved Sequences and Gaps in Alignment Chains

Orthologous pairwise alignment chains allow for convenient programmatic distinction between reference genome regions that are either intact and conserved at the orthologous position of the aligned query genome versus those that are missing or extensively diverged (Kent et al. 2003). We thus used the chains with gene ortholog mappings from the previous step to record single- and double-sided chain gaps (intervals predicted to be missing from the aligned genome relative to the reference) from each target species. To avoid mistaking incomplete genome assembly for genuinely missing sequence, we excluded (subtracted) chain gap intervals that are within 100 bp of an assembly gap longer than [*N*]_5_ in any given target genome. For each alignment chain, we merged any chain gap intervals that are within 20 bp of each other.

Using orthologous chains of outgroup species, we identified *conserved* genomic intervals by (a) recording regions in each query genome that exhibit an intact alignment block; (b) computing percent sequence identity relative to the oryLat04 reference using 10, 25, 50, and 100 bp sliding windows across the alignment blocks; (c) sorting the windows by percent sequence identity; and (d) keeping the top *M* most-conserved windows that, when merged and flattened, cover 5% of oryLat04, and together were deemed per-species *conserved elements*. For each alignment chain, we merged conserved elements within 20 bp of each other. Note that the conserved elements incorporate all additional windows with sequence identity scores tied with that of the *M*^th^ (lowest-scoring) window included to reach the 5% reference coverage threshold. Summary statistics for the sliding windows used to delineate conserved elements are in supplementary table S4, Supplementary Material online.

#### Screen for Genomic Regions Associated With Fin Reduction

For each Japanese Medaka reference gene that was successfully mapped to at least 17 pelvis-complete outgroups and 5 pelvis-reduced target species, we scanned the interval spanning 200 kb upstream and downstream of the canonical (longest) isoform's transcription start site (400,001 bp total). Within this interval, we identified *target deletions* by recording regions where sequence is missing (i.e., a valid chain gap is present) in at least ⅔ of all species in each of the two officially screened target clades (Tetraodontiformes and Syngnathidae). We then intersected the target deletions with regions that were covered by conserved elements in at least 17 outgroups (not including the reference assembly itself, which is sequence-conserved by definition) and that spanned at least 20 bp to identify raw candidate CONDELs. We merged raw CONDEL candidates within 20 bp of each other and only recorded final candidate intervals that are 50 bp or larger and that do not overlap a chain gap (i.e., have a genotype–phenotype violation) in more than one scorable outgroup chain. In this procedure, a CONDEL can be “called by” (linked to) multiple gene orthologs if they are less than 200 kb from the same candidate region. The set of pCONDEL-linked orthologs was used for the functional enrichment analysis described in the following section. In supplementary spreadsheet 2, Supplementary Material online, we report unique percomorph CONDEL intervals and note all linked orthologs that call a given candidate. CONDEL candidates (as well as reference gene annotations) can also be visualized at the following UCSC Genome Browser assembly hub (Kent et al. 2002; Raney et al. 2014): https://genome.ucsc.edu/s/hchen17/oryLat04_public.

#### pCONDEL Functional Enrichment Test

Using GO Ontology (Ashburner et al. 2000; The Gene Ontology Consortium 2021) Biological Process Complete annotations (DOI: 10.5281/zenodo.6799722 Released 2022-07-01) and false discovery rate multiple hypothesis testing correction, a PANTHER binomial overrepresentation test (Released 20220712) was performed on the 3,489 oryLat04 orthologs that yielded one or more pCONDEL candidates in the computational screen (Mi et al. 2019). All oryLat04 protein-coding genes (provided by PANTHER) constituted the background set for the binomial test. See supplementary spreadsheet 3, Supplementary Material online for a complete list of all ontology terms with *q*-value <0.05, all input genes, and all unmapped input genes (which are almost exclusively noncoding RNA genes).

#### Identifying CONDELs With Homology in the Zebrafish Genome

BLASTN (Altschul et al. 1997; version 2.7.1+; task specified to blastn; E-value threshold set to 1e−4; and word sizes set to 7 and 11) was used to search for homology between the Japanese Medaka sequence of each pCONDEL and the Zebrafish reference assembly danRer11 (GenBank accession # GCA_000002035.4). BLAST hits to chrUn and “alt” chromosomes were disregarded. In addition, we considered homology suggested by any pCONDEL conserved element that intersected (by at least 10 bp) an alignment block in the liftOver chains between oryLat04 and danRer11 (generated by doBlastzChainNet.pl as described above; Kent et al. 2003). All homology candidates were then manually inspected via UCSC Genome Browser visualizations of oryLat04 and danRer11 to verify orthology or paralogy for intragenic loci as well as preservation of genomic synteny for intergenic loci (Kent et al. 2002; Raney et al. 2014). Candidate regions that were identified by the initial BLAST and LASTZ alignment criteria above but that did not show convincing homology were deemed false positive hits and were noted accordingly in supplementary spreadsheet 2, Supplementary Material online (see column “conserved to zebrafish”).

#### Locating *PelA* in the Japanese Medaka Genome

BLASTN (version 2.7.1+) (task specified to blastn) was used to locate regions of sequence similarity between the Japanese Medaka reference genome sequence and the Threespine Stickleback 500 bp and 2.5 kb *PelA* enhancer sequences described by Chan et al. (Altschul et al. 1997; Chan et al. 2010).

#### Prediction of Transcription Factor Binding Sites Within CONDEL Candidates

The R/Bioconductor packages TFBSTools (version 1.34.0) and JASPAR2022 (version 0.99.7) were used to identify binding site predictions for all latest-version vertebrate transcription factors in the JASPAR 2022 database with a position weight matrix match score ≥925 (out of 1000) to either the forward or reverse complement of the Japanese Medaka CONDEL sequence (Tan and Lenhard 2016; Baranasic 2022; Castro-Mondragon et al. 2022). See script predictTFBS.R in code repository.

#### Generation of CONDEL Multiple Sequence Alignment From Orthologous Chains

The script getGappedMSAfromENSGmappedChains_v1.sh (see code repository) was used to extract each nonreference species’ orthologous sequence at the CONDEL coordinates from the associated pairwise alignment chains. Then, a gapped multiple sequence alignment was manually collated.

#### Phylogenetic Tree and Branch Length Calculation

To create a multiple sequence alignment for estimating the phylogenetic distance between species in the screen, we passed the pairwise netted alignments generated by doBlastzChainNet.pl into ROAST v3 (multiz) (Blanchette et al. 2004). We then used msa_view to identify and extract sufficient statistics for 4-fold degenerate sites in coding regions as defined by Ensembl release 98 ASM223467v1 gene models (Cunningham et al. 2022). We estimated the rate of neutral evolution at 4-fold degenerate sites using phyloFit (Hubisz, Pollard and Siepel 2011) under the REV substitution model, and used PhyloDM (Mussig 2022) to compute pairwise distances between all species in the screen (supplementary spreadsheet 4, Supplementary Material online). We based the percomorph phylogenetic tree topology on the consensus of several recent studies (see supplementary fig. S1 and text 1, Supplementary Material online) (Alfaro et al. 2018; Hughes et al. 2018; Mu et al. 2022). MEGA11 (version 11.0.13) was used to format and draw the resulting branch length-calibrated tree (Tamura, Stecher and Kumar 2021).

#### Nearest Outgroup Definition

To identify a given target clade's nearest outgroup for use in functional experiments, we considered the following factors in the listed order:

1. Proximity based on the most recent common ancestor by phylogenetic tree topology (fig. 1*A*)
2. Pairwise genetic distance based on the neutral model of evolution described above (see supplementary fig. S1, text 1, and file 4, Supplementary Material online)
3. Practical availability of high-quality genomic DNA or adequately preserved tissue samples

### Testing for Significant Phenotypic Differences Between Target and Outgroup Species

“Phylogenetically corrected” means for the numbers of pelvic fins and caudal rays in target and outgroup species were estimated using the phyloMean function of the MOTMOT R package (Thomas and Freckleton 2012; Puttick et al. 2019). Then, two-tailed Welch's *t*-tests were performed to determine if the traits differed significantly between target and outgroup species. See comparePhyloMeans.R script in the code repository.

### Percomorph Tissue and Genomic DNA Samples

Percomorph genomic DNA was isolated from ethanol-preserved fin, muscle, or liver tissue by incubation in lysis buffer (10 mM Tris, pH 8, 100 mM NaCl, 10 mM EDTA, 0.5% SDS, 500 μg/ml Proteinase K) at 55 °C for 4–12 h, followed by extraction with 25:24:1 phenol:chloroform:isoamyl alcohol, 1–2 washes in chloroform, ethanol precipitation, and resuspension of the DNA pellet in water or TE buffer. Sample and tissue sources are listed in supplementary table S2, Supplementary Material online.

### Species Verification by *Cytochrome Oxidase Subunit I* Sequencing

A segment of the mitochondrial gene *cytochrome oxidase subunit I* (*COI*) was amplified using DreamTaq Green PCR Master Mix (Thermo Scientific catalog # K1081) and primers percomorph-COI-fwd and percomorph-COI-rev (see supplementary table S5, Supplementary Material online), according to manufacturer recommendations. The subsequent Sanger sequencing reads were cross-referenced with the Barcode of Life Data System (http://v4.boldsystems.org/) to confirm species identity prior to further empirical interrogation of the genetic material (Ratnasingham and Hebert 2007). *COI* amplicon sequences of non-*Gasterosteus* species have been deposited in GenBank (see supplementary table S2, Supplementary Material online for accession numbers).

### Sequence Verification and Cloning of Candidate CONDEL Regions

We verified the computationally derived pCONDEL candidates near *Pitx1* and *Faf1* by PCR and Sanger sequencing to confirm the DNA sequence represented by the genome assemblies and alignments, according to the following procedure. To compare equivalent genomic sequence between species, we first identified—in the Japanese Medaka genome assembly—the nearest intervals upstream and downstream of the pCONDEL candidate that exhibit alignment blocks in nearly every outgroup *and* target species with a successful orthologous mapping to *Pitx1* or *Elavl4* and *Faf1*, respectively (see *Whole-genome Alignment and Orthology Mapping* methods above). We then used the coordinates of these flanking conservation anchors to extract comparable sequence from each query genome assembly using the script getConsAnchoredOrthologousFastas.sh (see code repository).

We designed amplification primers for the extracted assembly sequences using Geneious Prime (Biomatters Ltd) and generated PCR amplicons using Q5 High-Fidelity 2X Master Mix (New England Biolabs catalog # M0492), Phusion High-Fidelity PCR Master Mix with HF Buffer (New England Biolabs catalog # M0531), and/or LongAmp® Hot Start Taq DNA Polymerase (New England Biolabs catalog # M0534S) with Expand HF Buffer (Roche catalog # 05917131103) according to manufacturer recommendations. The PCR amplicons were then integrated into vector pCR4Blunt-TOPO using the Zero Blunt TOPO PCR Cloning Kit (Invitrogen catalog # 450031) for subsequent Sanger sequencing. Primers introducing homology arms for Gibson Assembly were used to clone candidate sequences into either the GFP reporter vector pT2HE (Howes, Summers and Kingsley 2017) and/or the firefly luciferase reporter vector pGL4.23[luc2/minP] (Promega, Genbank Accession # DQ904455.1) using NEBuilder HiFi DNA Assembly Master Mix (New England Biolabs catalog # E2621). Reporter constructs and candidate region amplicon sequences have been deposited in GenBank (see supplementary table S2, Supplementary Material online for accession numbers).

### Threespine Stickleback Husbandry

All Threespine Stickleback in this study were raised in 29-gallon tanks under standard aquarium conditions (3.5 g/L Instant Ocean salt, 18 ˚C) and fed live brine shrimp as larvae, then frozen daphnia, bloodworms, and/or mysis shrimp as juveniles and adults—in accordance with the Guide for the Care and Use of Laboratory Animals of the National Institutes of Health under Protocol #13834 of the Institutional Animal Care and Use Committee of Stanford University. All live fish in this study were lab-raised descendants of wild-caught fish. All quantitative in vivo experiments that tested specific hypotheses adhered to ARRIVE guidelines (Percie du Sert et al. 2020).

### Enhancer Reporter Assays in Transgenic Threespine Stickleback

Microinjection of freshly fertilized Threespine Stickleback eggs with *Tol2* transposase mRNA and GFP reporter constructs was performed as previously described (Chan et al. 2010; Thompson et al. 2018). All resulting larvae were raised under standard aquarium conditions to Swarup stage 30 (Swarup 1958) and then euthanized in 600 mg/L tricaine, pH 7.5 for phenotyping. GFP expression was documented using a Leica MZFLIII fluorescence microscope (Leica Microsystems) fitted with a GFP2 filter and ProgResCF camera (Jenoptik AG). Only fish exhibiting bilateral green eyes (from background expression by the *hsp70* minimal promoter of the expression construct, indicating extent of transgenesis) were phenotyped (Nagayoshi et al. 2008). The percomorph *PelA* constructs were assayed on lab-raised pelvic- and caudal-complete stickleback descended from fish collected at Rabbit Slough, Alaska, USA. The construct containing the Japanese Medaka ortholog of the *Faf1* CONDEL candidate region was assayed on lab-raised pelvic- and caudal-complete Threespine Stickleback descended from fish collected at Matadero Creek, California, USA and at Little Campbell River, British Columbia, Canada. The experiment did not include blinding, but post hoc PCR confirmation of integrated plasmid identity was performed.

### Luciferase Enhancer Reporter Assays

OLHNI-2, a Japanese Medaka fibroblast-like fin-derived cell line, was acquired from RIKEN BioResearch Resource Center (Cell No. RCB2942) and verified by sequencing a segment of the mitochondrial gene *COI* (see *Species Verification by* COI *Sequencing* above) (Hirayama, Mitani and Watabe 2006). The cells were cultured using Leibovitz's L-15 Medium (Gibco Catalog # 11415064) supplemented to 20% fetal bovine serum (ATCC Catalog # 30-2020) and 1 × penicillin–streptomycin (Gibco Catalog # 15140122), at 30 ˚C to 33 ˚C and ambient CO_2_ concentration. Firefly and pRL-SV40 renilla luciferase constructs (Promega, Genbank Accession # AF025845) were transfected using Solution SF (Lonza Catalog # V4SC-2096) and program DN-100 of the Amaxa Nucleofector 96-well Shuttle System according to manufacturer recommendations. Each firefly luciferase plasmid was independently amplified, miniprepped (ZymoResearch Catalog # D4210), and tested the following number of times: Marine Stickleback, 12; Yellow Croaker, 33; Green Spotted Puffer, 12; Ocean Sunfish, 8; European Seabass, 10; Gulf Pipefish, 11. Each plasmid preparation replicate was assayed in technical quadruplicate and reported as a single averaged point in figure 2*D*. For interexperiment normalization, aliquots of a single preparation of the basal firefly vector pGL4.23[luc2/minP] (Promega, Genbank Accession # DQ904455.1) were assayed on each experimental day. Dual-Luciferase Reporter Assay System reagents (Promega Catalog # E1910) and a Dual Injector System for GloMax-Multi Detection System (Promega) luminometer were used to measure luciferase activity. The plasmid locations on each plate were randomized to control for potential positional biases from the multichannel pipettes, nucleofector, and luminometer that were used. Two-tailed Mann–Whitney *U* tests were performed (using python package scipy v1.7.3) to compare the distribution of biological replicates (each the average of technical quadruplicates) between constructs, as this statistical test does not require the data to be normally distributed (Virtanen et al. 2020).

### RNAseq

A lab-raised Matadero Creek male Threespine Stickleback and lab-raised Little Campbell River female Threespine Stickleback were crossed to generate one clutch of wild-type F1 hybrid fish. Caudal fin buds were collected into 1.5 ml centrifuge tubes from 12 siblings of the clutch on 10 dpf (after hatching, before lepidotrichia development, Swarup stage 27) and 12 additional siblings on 17 dpf (during lepidotrichia development, Swarup stage 29) (Swarup 1958). The dissected tissues were immediately placed on dry ice and stored at −80 °C until RNA extraction was performed using the RNeasy Plus Micro Kit (Qiagen Catalog # 74034). Each fin bud was first homogenized in 50 µl of Buffer RLT (supplemented with ß-mercaptoethanol per kit recommendation) for 1 min at RT in a 1.5 ml centrifuge tube using a cordless motor (VWR Catalog # 47747-370) and pestle (USA Scientific Catalog # 1415-5390). The pestle was rinsed with an additional 300 µl of Buffer RLT into the 1.5 ml tube; then the resulting 350 µl volume was drawn twice into a 1 ml luer slip tip tuberculin syringe (BD Catalog # 309659) fitted with a 27G-½” needle. Each RNA sample was eluted in 16 µl of RNAse-free water and quantified using the Qubit RNA High Sensitivity Kit (Invitrogen Catalog # Q32852). A subset of the samples from each developmental stage was quality-checked by Bioanalyzer using the RNA 6000 Pico Kit (Agilent Technologies Catalog # 5067-1513). The resulting RNA integrity numbers were between 8.4 and 10, with most values 9.4 or higher.

Sequencing libraries were generated with the Stranded mRNA Prep kit (Illumina Catalog # 20040532) using 68–80 ng of RNA for 10 dpf samples and 150 ng of RNA for 17 dpf samples. The libraries were sequenced (2 × 150 bp) as a single multiplexed pool on two partial lanes of an Illumina NovaSeq 6000 by Novogene Corporation Inc. The resulting sequencing reads were trimmed with fastp v0.21.0 (Chen et al. 2018) and mapped to Threespine Stickleback genome assembly gasAcu1-4 via two-pass mapping with STAR v2.7.7a (Dobin et al. 2013) according to GATK best practices (Van der Auwera et al. 2013). The program featureCounts v2.0.1 (Liao, Smyth and Shi 2014) was used to quantify reads mapped per gene based on gasAcu1 gene annotations (Ensembl release 99) that were lifted to gasAcu1-4 as previously described (Roberts Kingman et al. 2021; Cunningham et al. 2022). Each sample's gene-level read counts were normalized to transcripts per million to assess expression levels of genes near *Faf1* at the two developmental stages (Li et al. 2010).

### CRISPR-Cas9 Genome Editing

Single-guide RNAs (sgRNAs) for directing Cas9 nuclease activity in Threespine Stickleback were designed using CHOPCHOP (Labun et al. 2016, 2019) and the genome assembly gasAcu1. Templates for the sgRNAs were synthesized via 2-oligo PCR and used for in vitro transcription by T7 RNA polymerase, as previously described (Wucherpfennig, Miller and Kingsley 2019). In addition to sgRNAs targeting candidate regions, an sgRNA for inactivating the *golden* pigment gene *Slc24a5* (Lamason et al. 2005) was also included as a visual control for editing status. Supplementary table S5, Supplementary Material online lists all oligonucleotides used to generate sgRNAs.

For recreating the pCONDEL.1189 candidate near *Faf1* in Threespine Stickleback, a 60 bp single-stranded DNA donor template was injected along with the sgRNAs and Cas9 protein. Intended for promoting homology-directed repair, this oligonucleotide was a concatemer of the two 30-mers on either side of the desired 464 bp deletion (see fig. 4*A* and supplementary table S5, Supplementary Material online), and the two most 5′ and two most 3′ bases were synthesized to have phosphorothioate (not phosphodiester) bonds to enhance stability in vivo (Integrated DNA Technologies, Inc).

Cas9-2NLS protein (QB3 MacroLab, University of California—Berkeley), sgRNAs, and single-stranded DNA oligonucleotides were injected at the indicated concentrations (supplementary table S3, Supplementary Material online) into freshly fertilized Threespine Stickleback eggs. For experiments in which half of each clutch was injected with reagents to target the pCONDEL.1189 region and the other half was injected with sgRNAs targeting only the *golden* locus, the order of injection was alternated between each of the 17 genome-edited clutches to control for any potentially confounding effects related to the time of injection after fertilization. To verify editing status in the resulting fish, dorsal spine and dorsal fin biopsies and/or skin mucus swabs were collected from fish after reaching ≥20 mm standard length and used for subsequent DNA extraction (Breacker et al. 2017). All phenotypic scoring was performed at or after fish reached 15 mm standard length, before any fin tissues were biopsied for genotyping; blinding was not performed. All CRISPR-Cas9 editing experiments were performed on either the Matadero Creek and/or Little Campbell River population background. Two-tailed, Boschloo's exact tests were performed (using python package scipy v1.7.3) to ascertain whether the genome editing was significantly associated with phenotypic outcome, as this statistical test is conditioned only on one marginal sum (the number of fish in each treatment or control group) (Boschloo 1970; Mehrotra, Chan and Berger 2003; Ludbrook 2013; Virtanen et al. 2020).

**Sample size calculations:** ​​A pilot experiment to target the pCONDEL.1189 candidate region in Threespine Stickleback from Matadero Creek suggested the ectopic caudal ray phenotype occurred in ∼5% of injected individuals—and the controls for this pilot experiment consisted of unmodified clutch siblings, which all developed wild-type caudal fins. After completing the pilot experiment, we incidentally observed that a spontaneous caudal fin morphology reminiscent of the pCONDEL.1189-edited phenotype appears at very low frequencies (∼0.5%) in wild-type, unmodified Threespine Stickleback from Matadero Creek. Given this finding, we performed power calculations (alpha = 0.05, beta = 0.2, power = 0.8) to determine the appropriate sample size for a replication experiment in which control siblings would also be injected to edit the unrelated *golden* pigment locus to account for possible confounding effects from introducing Cas9 nuclease into the zygote. We report full results of the replication experiment here (involving 17 genome-edited clutches total; see supplementary fig. S5, Supplementary Material online). Because the *golden*-edited controls from the replication experiment above did not suggest confounding effects from the presence of Cas9 nuclease, the subsequent experiment targeting coding exons of *Faf1* used unmodified clutch siblings as controls. A pilot experiment to knockout *Faf1* yielded a similar (∼5%) frequency of ectopic caudal ray phenotypes, so we aimed to achieve a similar sample size as above to test the effect of *Faf1* inactivation in a subsequent replication experiment (involving 10 genome-edited clutches total; see supplementary fig. S8, Supplementary Material online).

### Primers and DNA Oligonucleotides

Sequences for all DNA oligonucleotides and genotyping primers are listed in supplementary table S5, Supplementary Material online.

### Skeletal Preparations

Adult Threespine Stickleback (≥30 mm standard length), a Green Spotted Pufferfish, and a juvenile Rice Eel were fixed in 10% neutral buffered formalin for at least 5 days, rinsed twice in distilled water and twice in 30% saturated sodium borate, and then cleared in a solution of 1% porcine trypsin dissolved in 30% saturated sodium borate for 24–48 h at RT. After equilibrating the cleared specimens to 2% potassium hydroxide (KOH), staining of calcified skeletal tissue was performed by incubation in 0.005% Alizarin Red S dissolved in 2% KOH for 12–24 h; and then pigment was bleached by incubating the fish in 0.375% KOH, 25% glycerol, and 0.0003% hydrogen peroxide. Juvenile Threespine Stickleback (≤20 mm standard length) that required genotyping after skeletal characterization were stained using Walker and Kimmel's two-color acid-free method (at 100 mM magnesium chloride concentration) (Walker and Kimmel 2007). Finally, all specimens were passed through a 0.375% KOH:glycerol gradient before imaging and storage in 100% glycerol. An Epson Perfection V800 Photo scanner and a Stemi SV11 stereomicroscope fitted with an AxioCam HRc camera (Carl Zeiss AG), respectively, were used to record brightfield images of adult and juvenile skeletal preparations.

### Code, Data, and Material Availability

A code repository with instructions for replicating the computational screen starting with flat-text genome assembly fasta files is available at https://github.com/bejerano-lab/percomorphCONDELs. To aid users who may not have access to a compute cluster, parts of the screen were implemented to optionally use GNU Parallel (Tange 2011). Supplementary spreadsheet 1, Supplementary Material online lists genome assembly accession identifiers and sources. A copy of precomputed, genome assembly-derived intermediate inputs (including whole-genome alignment chains) for generating the final candidate list has been deposited at https://doi.org/10.5281/zenodo.7140838. Gene annotations and pCONDEL candidate regions in the Japanese Medaka reference genome can be viewed at the following UCSC Genome Browser assembly hub (Kent et al. 2002; Raney et al. 2014): https://genome.ucsc.edu/s/hchen17/oryLat04_public. A copy of the files underlying the Japanese Medaka assembly hub is available at https://doi.org/10.5281/zenodo.7909719. Supplementary table S2, Supplementary Material online lists the source of percomorph tissue and genomic DNA used in this study as well as the GenBank accession numbers for the derived DNA sequences and constructs. Raw RNA-seq data are available through National Center for Biotechnology Information (NCBI) BioProject number PRJNA908888. Other materials will be made available upon reasonable request.

## Supplementary Material

msad188_Supplementary_Data
